# Silver Covalently Bound to Cyanographene Overcomes Bacterial Resistance to Silver Nanoparticles and Antibiotics

**DOI:** 10.1002/advs.202003090

**Published:** 2021-05-03

**Authors:** David Panáček, Lucie Hochvaldová, Aristides Bakandritsos, Tomáš Malina, Michal Langer, Jan Belza, Jana Martincová, Renata Večeřová, Petr Lazar, Kateřina Poláková, Jan Kolařík, Lucie Válková, Milan Kolář, Michal Otyepka, Aleš Panáček, Radek Zbořil

**Affiliations:** ^1^ Regional Centre of Advanced Technologies and Materials Czech Advanced Technology and Research Institute Palacký University Olomouc Křížkovského 511/8 Olomouc 779 00 Czech Republic; ^2^ Department of Physical Chemistry Faculty of Science Palacký University Olomouc 17. listopadu 1192/12 Olomouc 771 46 Czech Republic; ^3^ Regional Centre of Advanced Technologies and Materials Palacký University Olomouc Šlechtitelů 27 Olomouc 783 71 Czech Republic; ^4^ Nanotechnology Centre Centre of Energy and Environmental Technologies VŠB–Technical University of Ostrava 17. listopadu 2172/15 Ostrava‐Poruba 708 00 Czech Republic; ^5^ Department of Microbiology Faculty of Medicine and Dentistry Palacký University Olomouc Hněvotínská 3 Olomouc 775 15 Czech Republic

**Keywords:** antimicrobial, cytocompatibility, graphene, silver resistant

## Abstract

The ability of bacteria to develop resistance to antibiotics is threatening one of the pillars of modern medicine. It was recently understood that bacteria can develop resistance even to silver nanoparticles by starting to produce flagellin, a protein which induces their aggregation and deactivation. This study shows that silver covalently bound to cyanographene (GCN/Ag) kills silver‐nanoparticle‐resistant bacteria at concentrations 30 times lower than silver nanoparticles, a challenge which has been so far unmet. Tested also against multidrug resistant strains, the antibacterial activity of GCN/Ag is systematically found as potent as that of free ionic silver or 10 nm colloidal silver nanoparticles. Owing to the strong and multiple dative bonds between the nitrile groups of cyanographene and silver, as theory and experiments confirm, there is marginal silver ion leaching, even after six months of storage, and thus very high cytocompatibility to human cells. Molecular dynamics simulations suggest strong interaction of GCN/Ag with the bacterial membrane, and as corroborated by experiments, the antibacterial activity does not rely on the release of silver nanoparticles or ions. Endowed with these properties, GCN/Ag shows that rigid supports selectively and densely functionalized with potent silver‐binding ligands, such as cyanographene, may open new avenues against microbial resistance.

## Graphical abstract

Silver covalently bound to cyanographene sheets kills persistent bacteria, which are resistant to silver nanoparticles, at trace level concentrations. Owing to the strong and multiple dative bonds between the nitrile groups of cyanographene and silver, there is minimum silver ion leaching, leading eventually to an exceptional combination of antibacterial activity with cytocompatibility.

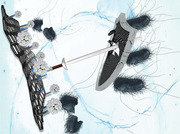

## 1 Introduction

Antimicrobial resistance threatens the very core of modern medicine,^[^ ^1^ ^]^ undermining the humankind's discoveries of the last century against many routinely treated bacterial infections. According to a 2016 report by the United Nations General Assembly, it may be estimated that if bacterial resistance continues to grow at the same rate, untreatable infections caused by multidrug‐resistant bacteria will become the primary cause of death by 2050.^[^ ^2^ ^]^ It is therefore vital to adequately address this issue systematically, or the probability of returning to the pre‐antibiotic era, when a simple infection was fatal, may alarmingly increase.^[^ ^3^ ^]^

Inorganic^[^ ^4^, ^5^, ^6^, ^7^, ^8^, ^9^ ^]^ and carbon‐based nanomaterials,^[^ ^9^, ^10^, ^11^, ^12^ ^]^ polymers and peptides,^[^ ^13^, ^14^ ^]^ as well as light‐activated nanomaterials^[^ ^15^, ^16^ ^]^ have emerged as promising antimicrobial agents for treatment and prevention of infectious diseases. Particularly silver colloids can inhibit growth of pathogens at very low concentrations.^[^ ^17^, ^18^, ^19^ ^]^ However, the development of resistance even to silver nanoparticles (AgNPs) was demonstrated,^[^ ^20^ ^]^ whereby bacteria started to secret a protein (flagellin) which induced coagulation of the AgNPs and reduced dramatically their antibacterial activity. Only after administration of additional molecular substances the release of flagellin was blocked and AgNPs restored their antibacterial activity. These results highlight the risk of entering another race for the discovery of anti‐flagellin substances faster than the development of resistance from bacteria to them. Although methods to increase colloidal stability of AgNPs via surface modification have been applied to prevent aggregation and preserve antibacterial activity, they were insufficient against flagellin‐induced aggregation.^[^ ^20^ ^]^ Graphene oxide (GO) has been used as a rigid support for AgNP immobilization to bypass aggregation,^[^ ^10^, ^11^, ^12^, ^21^, ^22^, ^23^ ^]^ but its surface is chemically inhomogeneous with many different oxygen‐containing groups,^[^ ^24^, ^25^ ^]^ preventing a strong and selective surface chemistry for silver binding. Furthermore, according to the hard‐soft acid‐base theory, oxygen functionalities are poor coordination ligands for silver.^[^ ^26^, ^27^ ^]^

**Figure 1:**
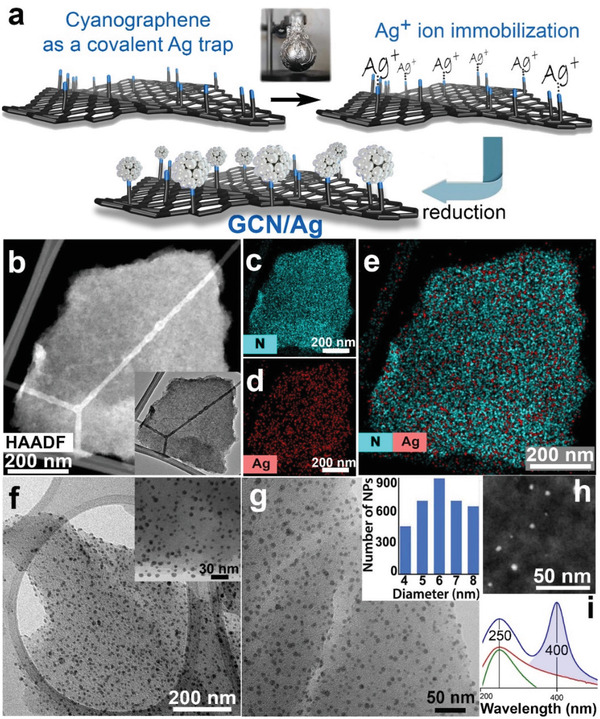
a) Reaction scheme for the preparation of silver nanoparticles bonded on the nitrile groups of cyanographene (GCN/Ag). b) HAADF‐STEM image (and TEM image, inset) of a GCN flake after interaction with AgNO_3_. EDS chemical mapping of c) nitrogen and d) silver. e) Combined chemical mapping of nitrogen and silver on the flake shown in panel (b). f,g) TEM images of GCN/Ag and size distribution of the AgNPs (inset in panel (g)). h) HAADF‐STEM image of GCN/Ag showing the AgNPs as bright spots. i) Light absorption spectra of the starting GCN (bottom green curve), the GCN/Ag^+^ precursor (middle red curve), and after reduction, the GCN/Ag product (top blue curve).

To tackle such issues, we used a densely functionalized graphene (cyanographene, GCN^[^ ^28^ ^]^), which proved a very efficient covalent trap for silver ions, exploiting the high coordination proclivity of nitrile groups toward silver.^[^ ^26^, ^27^ ^]^ The trapping of single Ag ions allowed the high‐quality purification of the GCN/Ag^+^ precursor and the subsequent reduction of only those Ag ions that remained coordinated on GCN (**Figure** **1**a, and Methods in the Supporting Information). The strong covalent immobilization afforded a material with groundbreaking properties: i) potent antibacterial activity, similar to free ionic silver, even against multidrug‐resistant bacterial strains, ii) minimum bactericidal concentrations against AgNP‐resistant bacterial strains 30‐fold lower than free AgNPs (benchmarked under identical conditions), and iii) very low leaching of silver ions or AgNPs, ascribing very high cytocompatibility to healthy human cells, which is a very critical asset for practical applications.

## 2 Results and Discussion

The GCN/Ag^+^ precursor (prepared in the dark, Figure 1a) comprised flakes of GCN free from AgNPs, as high‐angle annular dark‐field scanning transmission electron microscopy (HAADF‐STEM) imaging revealed (Figure 1b). Higher resolution images of the ionic GCN/Ag^+^ precursor (Figure S1, Supporting Information) further confirmed the absence of AgNPs and elemental chemical mapping (Figure 1c–e) evidenced the dense and homogeneous coverage of the flakes by Ag, as well as by the nitrogen atoms of the nitrile groups. After removing any unbound silver ions by thorough washing, reduction with NaBH_4_ afforded the final GCN/Ag product, comprising small AgNPs (Figure 1f–h) with diameter from 4 to 8 nm (Figure 1g, inset). Optical absorption of the GCN/Ag^+^ precursor and of GCN/Ag revealed the characteristic surface plasmon resonance of metallic AgNPs at 400 nm^[^ ^29^ ^]^ only after the reduction (Figure 1i), verifying the synthetic pathway (the full UV‐vis. absorption spectra are available in Figure S3, Supporting Information). The Ag content in the hybrid was 13 wt%, according to atomic absorption spectroscopy analysis. A control experiment with GO, following the same synthetic protocol, resulted in large size variations of the grown AgNPs with irregular topological distribution (Figure S2, Supporting Information), highlighting the role of the GCN support.

**Figure 2:**
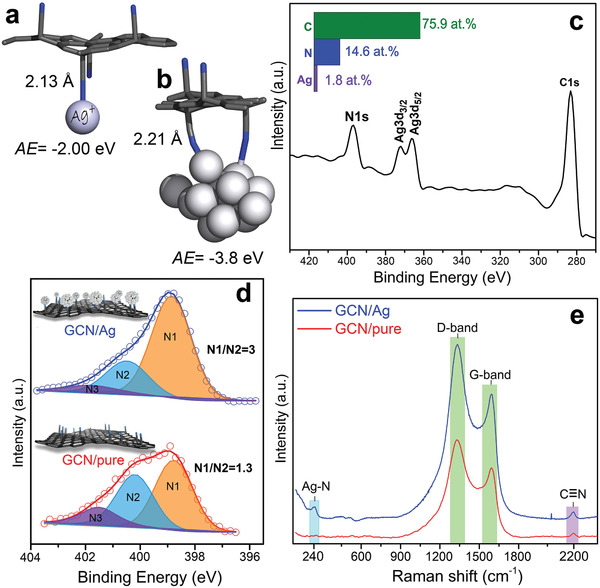
Theoretical models of GCN interacting a) with one silver cation and b) with a silver nanoparticle; 2.21 Å corresponds to the shorter bond. c) XPS survey spectrum of the GCN/Ag. d) Deconvoluted N1s HR‐XPS of the starting GCN and the GCN/Ag product. e) Raman spectra for GCN and GCN/Ag.

Theoretical calculations confirmed the strong immobilization of Ag^+^ ions on GCN with adsorption energy (AE) of −2.00 eV, indicating bond formation between the Ag^+^ ion and the N atom of the nitrile groups (**Figure** **2**a). Electron localization function of the Ag—N bond remained localized on individual atoms (Figure S4, Supporting Information). However, Mulliken and Hirshfeld charge analyses showed significant charge transfer from GCN to the 5s orbitals of Ag^+^ resulting in the fractional charge of 0.5 *e* on the Ag ion. Therefore, the Ag—N bond can be characterized as a strongly polarized covalent bond. The calculated bond length of 2.13 Å was in line with a typical N—Ag coordination bond (2.1–2.4 Å).^[^ ^30^, ^31^ ^]^ When Ag atoms aggregated into metallic AgNPs, the *AE* strengthened (−3.80 eV) owing to multiple bonding (Figure 2b). Silver donated electrons to GCN, because the Hirshfeld partial charge was +0.51 *e* on the AgNP, from which 0.19 *e* was localized on the silver atom bonded to nitrogen. Considering the size of the AgNPs and the coverage density of the CN groups on graphene (≈14%), it is plausible that each AgNP can establish several bonds to the nitriles and, therefore, attach very strongly to GCN (a GCN area of 10 × 14 Å may contain five nitrile groups on one side, with a mean distance of less than 1 nm).

The predicted strong interactions were verified experimentally with high‐resolution X‐ray photoelectron (HR‐XPS) and Raman spectroscopies. XPS showed the overall composition from carbon, nitrogen, and silver (Figure 2c), while the N1s region revealed intriguing area redistribution of the N1 and N2 components after immobilization of silver (Figure 2d). In particular, the area of the lower binding energy (BE) N1 component increased significantly at the expense of the higher BE N2 component, reflecting an increase of the electron density of N atoms after their bonding with metallic silver. This was in agreement with the electron donation from AgNPs identified from the calculations, and with previous reports on BE reduction of N or O upon interaction with AgNPs.^[^ ^32^, ^33^ ^]^ Raman spectroscopy (Figure 2e) more clearly confirmed such a N—Ag bonding, by the appearance of the band at 240 cm^−1^.^[^ ^34^ ^]^ Theoretical calculations (see Methods and Computations in the Supporting Information) indeed showed a frequency for the N—Ag stretching vibration at 230 cm^−1^. The nitrile groups were also evident in Raman and in Fourier transform infrared (FTIR) before and after AgNPs immobilization (Figure S5, Supporting Information), indicating their preservation after the reaction. The strong bonding was probably responsible for the formation of uniform and small‐diameter AgNPs, unlike the case of the control experiment with GO (Figure S2, Supporting Information).

**Figure 3:**
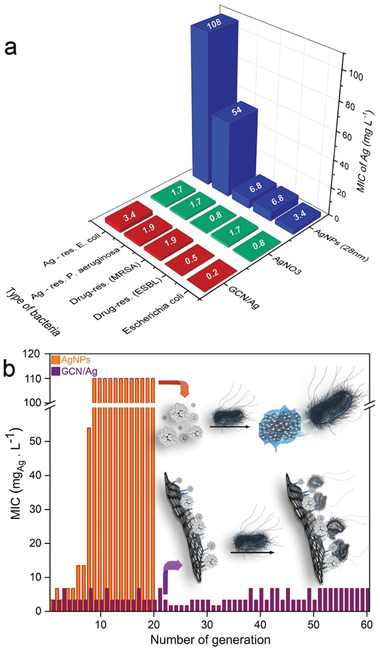
a) Comparative graph of MIC_100_ values for GCN/Ag, colloidal silver nanoparticles (AgNPs) and ionic silver (AgNO_3_) for different bacterial strains. MIC_100_ values of GCN/Ag refer to the Ag content only, for appropriate comparison with AgNO_3_ and AgNPs. In Table S2 (Supporting Information), MIC_100_ values with respect to the total GCN/Ag mass are also available. ^a)^MRSA: methicillin‐resistant *S. aureus*; ^b)^ESBL: extended‐spectrum *β*‐lactamases producing *Klebsiella pneumoniae*. MIC_100_ values were determined according to the European Committee on Antimicrobial Susceptibility Testing,^[^ ^42^ ^]^ as described in the section Methods in the Supporting Information. MIC_100_ for GCN/Ag with error bars is available in Figure S6 (Supporting Information). b) *E. coli* treated for several generations (serial passages) at subinhibitory concentrations with the GCN/Ag hybrid (violet) and with colloidal AgNPs (orange). Bacteria developed resistance and inactivated AgNPs, but not GCN/Ag. The serial passages with colloidal AgNPs were performed in the frame of a previous publication^[^ ^20^ ^]^ from some of the authors of this work; here these data are plotted for the first time.

Recently, Panacek et al. reported that Gram‐negative bacteria (which are increasingly becoming untreatable by modern antibiotics)^[^ ^35^ ^]^ can develop resistance even to initially highly active AgNPs.^[^ ^20^ ^]^ Exposure of 20 bacterial generations to subinhibitory concentrations of AgNPs induced flagellin production and aggregation/deactivation of AgNPs.^[^ ^20^ ^]^ Therefore, bacterial resistance even to AgNPs poses a serious threat. While the antibacterial activity of silver and silver composites range at quite low minimum inhibitory concentrations (MIC), i.e., 0.2–3.4 mg_Ag_ L^−1^ (Tables S1 and S2, Supporting Information), there are no reports for antibacterial agents against AgNP‐resistant bacteria. Studies against ionic Ag^+^‐resistant strains, mediated by the Ag^+^ efflux pump, reported MIC for AgNPs of 70 mg_Ag_ L^−1^.^[^ ^36^ ^]^ With the focus on addressing the alarming implications of bacterial resistance,^[^ ^3^ ^]^ GCN/Ag was evaluated against antibiotic‐susceptible, but also against multidrug‐ and AgNP‐resistant bacteria (AgNP‐resistant *Escherichia coli* and AgNP‐resistant *Pseudomonas aeruginosa* were developed as recently reported;^[^ ^20^ ^]^ see Methods in the Supporting Information for detailed description of the bacterial strains and Table S2 (Supporting Information) for the detailed results for the eight tested bacterial strains). As shown in **Figure** **3**a and Table S2 (Supporting Information), the MIC_100_ (i.e., MIC for 100% growth inhibition) values of GCN/Ag ranged at ultralow levels, from 0.2 to 7.2 mg_Ag_ L^−1^ (or 1.8–59.7 mg L^−1^ with respect to the total GCN/Ag mass), while pure GCN and GO did not show any antibacterial activity at concentration as high as 1880 and 1500 mg L^−1^, respectively (Table S2, Supporting Information). AgNPs of 28 and 10 nm diameter were synthesized and evaluated under similar testing conditions. The MIC_100_ values of GCN/Ag against several bacterial strains were lower than 28 nm AgNPs (Figure 3a) and similar to those of ionic silver (Figure 3a) or 10 nm AgNPs (Table S2, Supporting Information). Interestingly, they remained highly potent even against severely resistant strains, such as extended‐spectrum *β*‐lactamase (ESBL)‐producing *K. pneumoniae* ^[^ ^37^ ^]^ and methicillin‐resistant *S. aureus*.^[^ ^38^ ^]^ Impressively, GCN/Ag was ≈30 times more effective against AgNP‐resistant bacteria than both 28 and 10 nm colloidal AgNPs and similar to AgNO_3_ (Figure 3a and Table S2, Supporting Information). However, free silver ions are severely limited by their generic toxicity^[^ ^39^ ^]^ and are subjective to the resistance mechanisms which microorganisms developed during their 3–4 billion years of natural evolution and occasional exposure to toxic metal‐rich environments.^[^ ^26^ ^]^ To unequivocally prove the persistence of the high antibacterial activity of GCN/Ag, serial passages^[^ ^40^ ^]^ were performed for 60 *E. coli* bacterial generations (Figure 3b). The MIC_100_ for GCN/Ag increased only marginally, from 3.4 to 7 mg L^−1^. When the same bacteria were treated with conventional colloidal AgNPs under the exact same conditions, *E. coli* developed resistance on the 20th generation from 3.4 to ≈108 µg mL^−1^ (Figure 3b). These results verified our hypothesis that the very strong binding of silver on GCN can bypass the key resistance mechanism (induction of aggregation) of these bacteria against AgNP colloids. GCN/Ag appears to open the doors to a so far unmet challenge, bypassing the bacterial resistance mechanisms of some of the most threating microorganisms, such as *E. coli* and *P. aeruginosa*.^[^ ^41^ ^]^

**Figure 4:**
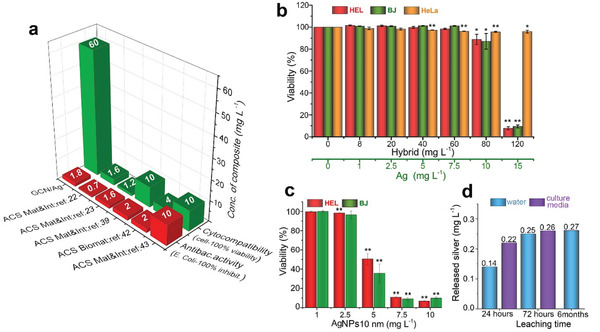
a) Comparative graph of the antibacterial activity and cytocompatibility of GCN/Ag in healthy human cells compared to representative examples from literature; in the latter case obtained on human cancer cell lines. Extended comparisons are also available in Table S1 (Supporting Information). b) Viability of human lung fibroblasts *HEL*, human skin fibroblasts *BJ*, and cancer HeLa cells treated with GCN/Ag, expressed in terms of hybrid (black line) and in terms of silver content (green line) (*n* = 3. c) Viability of *HEL* and *BJ* cells (*n* = 3) treated with 10 nm AgNPs. d) Leaching test of silver from GCN/Ag in water and in cell‐culture medium after 24, 72 h, and six months. The concentrations on the columns correspond to 0.07%, 0.11%, 0.13%, 0.13%, and 0.14% of Ag leached from the total amount of Ag (200 mg L^−1^ of Ag) that was initially contained in GCN/Ag which was added in the solution for the leaching test. *p ≤ 0.05; **p ≤ 0.01.

Considering the applicability of antimicrobial agents, their biocompatibility is an equally important asset, as silver exerts a generic cytotoxic effect.^[^ ^43^ ^]^ Therefore, the cytocompatibility of GCN/Ag was investigated with flow cytometry (using propidium iodide and calcein fluorescent probes, Supporting Information) on human skin fibroblasts, because of the potential application of antibacterial agents on skin, and on human lung fibroblasts (HEL 12469) for further establishment of the cytocompatibility profile. It was very gratifying to observe that GCN/Ag was fully tolerated by both cell lines up to 60 mg L^−1^ (or 7.5 mg_Ag_ L^−1^, **Figure** **4**a,b), which was ≈4–37 times higher than its antibacterial MIC_100_ values (Figure 3a). Such a high cytocompatibility combined with potent antibacterial activity against multidrug‐resistant strains and, strikingly, even against AgNP‐resistant strains, may introduce new thrust in the field. This is also evident by the comparisons in Figure 4a, showing that the cytocompatibility of GCN/Ag is significantly better than that of other graphene/silver hybrids with similarly potent antibacterial activities.^[^ ^22^, ^23^, ^39^, ^44^, ^45^ ^]^ These works were selected because of their very low MIC_100_ values and of the fine distribution of small AgNPs on the graphene sheets. It should be noted though, that in most of the reports, cancer cells (HeLa) were commonly used,^[^ ^39^, ^44^, ^45^ ^]^ which are significantly more tolerant to Ag than the healthy cell lines (Figure 4b). The latter were used in this study, as a more rigorous evaluation method. More comparisons with literature are available in Tables S1 and S3 (Supporting Information), where the differences in cell lines are also reported. The high cytocompatibility of GCN/Ag was further demonstrated by the comparison with 10 nm AgNPs colloids, whose cytocompatibility was limited to 2.5 mg_Ag_ L^−1^ (Figure 4c), as opposed to the 7.5 mg_Ag_ L^−1^ for the case of GCN/Ag (Figure 4b). Unequivocally, the high safe dose is the second key benefit of GCN/Ag, probably stemming from the strong bonding of silver on the surface of GCN.

The robust immobilization of silver on GCN was experimentally supported by TEM measurements of a GCN/Ag dispersion in water after six months of storage (Figure S7, Supporting Information), whereby immobilized AgNPs fully retained their original shape and size. Leaching tests for released silver further substantiated the strong binding, as after 72 h of storage in water or in cell culture media, leaching of silver reached 0.26 mg L^−1^ (Figure 4d), well below the toxic levels of GCN/Ag (10–15 mg_Ag_ L^−1^, Figure 4b) or of 10 nm AgNPs colloids (≈5 mg L^−1^, Figure 4c). The leached amount of Ag corresponded to 0.14% from the total amount of Ag initially contained in GCN/Ag which was added in the solution for the leaching test. Even after six months of storage in water, leaching remained practically the same (0.27 mg L^−1^ or 0.14%). To investigate further the release of silver, the MIC_100_ values of GCN/Ag were compared with free AgNPs and Ag^+^ ions with and without the addition of a silver‐ion complexing molecule^[^ ^46^ ^]^ (thioglycolate, NATG, Table S4, Supporting Information). Results showed that MIC_100_ values significantly increased in presence of NATG only for the case of AgNO_3_ (16 times) and for AgNPs (eight times), while for the case of GCN/Ag, the MIC_100_ increased only four times. Although this increase can also be affected by the binding of NATG on the AgNPs themselves, the comparative results corroborate the minor role of released Ag^+^ ions from GCN/Ag and its different mechanism of action.

**Figure 5:**
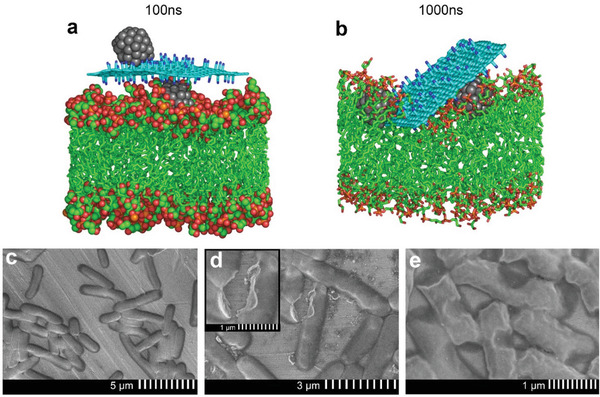
Snapshots taken from MD simulation at a) 100 ns and b) 1.0 µs show the interaction of GCN/Ag with the phospholipid membrane. More snapshots are shown in Figures S9 and S10 (Supporting Information) (color coding: cyan and green – carbon; red – oxygen; blue – nitrogen; gray – silver; orange – phosphorus, water molecules, ions, and hydrogen atoms are omitted for clarity); c) SEM image of native *E. coli* and d,e) treated with GCN/Ag at subinhibitory concentration (0.2 mg mL^−1^).

For better understanding the GCN/Ag–bacterial interface, we modeled by molecular dynamics (MD) simulations the interactions of GCN/Ag with a simplified model of bacterial plasma membrane consisting of a double layer of negatively charged 1‐palmitoyl‐2‐oleoyl‐sn‐glycero‐3‐phosphoglycerol (POPG) lipids (see the Supporting Information for more details). The hybrid stayed in contact with the membrane floating flat on its surface (**Figure** **5**a) for 0.1 µs without any sign of desorption, demonstrating a high affinity of the GCN/Ag to the membrane. Progressively (Figure S8, Supporting Information), GCN/Ag submerged into the polar headgroup region of POPG after 1 µs (Figure 5b), penetrating only slightly the hydrophobic part of the membrane, but generating a significant perturbation to its structure. MD simulations of GCN and AgNPs alone (Figure S9a,b, Supporting Information) also showed a very small extent of penetration to the hydrophobic membrane; both GCN/Ag and AgNPs were partly covered with the polar head groups (red spheres) of the lipids. On the contrary, MD simulations with graphene showed full penetration in the hydrophobic membrane compartment (Figure S9c, Supporting Information). Additional MD simulation of a mixed membrane consisted of 1‐palmitoyl‐2‐oleoyl‐sn‐glycero‐3‐phosphoethanolamine (POPE):POPG in the proportion 3:1 demonstrated the same behavior as the simulation with homogeneous POPG membrane (Figures S9 and S10, Supporting Information). The above results indicated that the antibacterial activity of GCN/Ag initiates on the extracellular level, as the internalization of the whole hybrid entities is less probable owing to the strong interactions with the outer membrane layer of the cell walls.

Certainly, the binding of AgNPs, or hybrids thereof, on the cell membrane can cause a cascade of events, culminating in degradation of the cell function and production of reactive oxygen species,^[^ ^43^, ^47^ ^]^ as it was also confirmed in the present case (Figure S11, Supporting Information). It is known that AgNPs bind to —SH groups of cell‐membrane proteins, altering their structure and function.^[^ ^17^ ^]^ They also interact with the proteoglycan‐rich bacterial biofilm, inhibiting its formation^[^ ^48^ ^]^ and altering proteoglycan expression.^[^ ^49^ ^]^ It is indicative that in the case of the Gram‐positive bacteria, tested in the present work (Table S2, Supporting Information), which express a proteoglycan extracellular matrix, GCN/Ag remained potent (Table S2, Supporting Information). Membrane‐wall damage has been suggested as a result of AgNPs binding (direct or indirect it is not known). For instance, *E. coli* were treated with subinhibitory concentration of AgNP colloids, and scanning electron microscopy (SEM) showed the formation of pits on the bacterial walls.^[^ ^50^ ^]^ In the present case as well, SEM characterization of *E. coli* incubated in absence (Figure 5c) and presence of GCN/Ag at subinhibitory concentrations (Figure 5d,e), whereby the bacterial population remains alive, also revealed significant membrane damage. The observed pits were rather severe in comparison to the previous report,^[^ ^50^ ^]^ despite the much lower Ag concentration which was used in our case (0.2 mg L^−1^). In the case of different antibacterial agents (i.e., carbon dots), the membrane walls presented very different morphology.^[^ ^51^ ^]^ Lack of significant wall damage in *E. coli* was also observed after treatment with antibacterial peptides^[^ ^52^ ^]^ and natural antimicrobial molecular agents.^[^ ^53^, ^54^ ^]^ Therefore, the particularly defective shape of alive *E. coli* cells observed in the present case could be ascribed to the action of GCN/Ag. SEM analyses on AgNP‐resistant *E. coli* and on multiresistant *S. aureus* are also available in Figures S12 and S13 (Supporting Information). It will be interesting to unveil in future the effects of protein binding of AgNPs that are already firmly grafted on a substrate (as in GCN/Ag). In such a case, the proteins’ motion and function might be more restricted than when bound to free/colloidal AgNPs. This hypothesis becomes more intriguing considering that bacteria require considerably higher membrane fluidity for normal growth and function^[^ ^55^, ^56^ ^]^ than eukaryotic cells,^[^ ^57^ ^]^ a matter that could also be related to the lower toxicity of the GCN/Ag to human cell lines.

## 3 Conclusions

In this work, a densely and selectively functionalized graphene was used as a trap for silver, exploiting its strong coordination with the nitrile groups of GCN. The binding energies approached values of covalent bonding, even surpassing them in case of multiple binding of one AgNP to several —CN groups, owing to the dense and homogeneous functionalization of GCN. This work also shows that bacteria which have developed resistance to AgNPs are highly susceptible on GCN/Ag. The persistence of the antibacterial activity was verified during serial passages over 60 bacterial generations (with no evidence of resistance development from the bacteria), while colloidal AgNPs lost their activity after 20 generations. Another key feature of GCN/Ag, critical for practical applications, was its very high cytocompatibility to healthy human cells in comparison to other reported hybrids, free AgNP colloids, and silver ions. This was ascribed to the strong GCN–silver interactions, which profoundly suppressed silver leaching, as theoretical calculations, modeling, and experiments confirmed. The present findings open the way to promising broad‐spectrum antibacterial agents, bypassing known resistance mechanisms of microorganisms.

## Conflict of Interest

The authors declare no conflict of interest.

## Supporting information

Supporting Information

## Data Availability

The data that support the findings of this study are available from the corresponding author upon reasonable request.
