# Supplementary material for: Silver Covalently Bound to Cyanographene Overcomes Bacterial Resistance to Silver Nanoparticles and Antibiotics
Source: Adv Sci (Weinh). 2021 May 3;8(12):2003090. doi: 10.1002/advs.202003090 (PMC8224420; doi:10.1002/advs.202003090)
Supplement: Supplementary file 1 — Supporting Information [file ADVS-8-2003090-s001.pdf]

© 2021 Wiley-VCH GmbH

ADVANCED SCIENCE
Open Access

Supporting Information

for *Adv. Sci.*, DOI: 10.1002/advs.202003090

Silver Covalently Bound to Cyanographene Overcomes Bacterial Resistance to Silver Nanoparticles and Antibiotics

David Panáček, Lucie Hochvaldová, Aristides Bakandritsos, Tomáš Malina, Michal Langer, Jan Belza, Jana Martincová, Renata Večeřová, Petr Lazar, Kateřina Poláková, Jan Kolařík, Lucie Válková, Milan Kolář, Michal Otyepka, Aleš Panáček,* and Radek Zbořil**

© 2020 Wiley-VCH GmbH

Supporting Information

Silver Covalently Bound to Cyanographene Overcomes Bacterial Resistance to Silver Nanoparticles and Antibiotics

David Panáček, Lucie Hochvaldová, Aristides Bakandritsos, Tomáš Malina, Michal Langer, Jan Belza, Jana Martincová, Renata Večeřová, Petr Lazar, Kateřina Poláková, Jan Kolařík, Lucie Válková, Milan Kolář, Michal Otyepka, Aleš Panáček,* and Radek Zbořil**

Dr. A. Bakandritsos, L. Hochvaldová, Dr. T. Malina, J. Belza, Dr. P. Lazar, J. Martincová, Dr. J. Kolařík, L. Válková, Prof. M. Otyepka, Dr. A. Panáček
Regional Centre of Advanced Technologies and Materials, Palacký University Olomouc, Šlechtitelů 27, 783 71 Olomouc, Czech Republic
E-mails: a.bakandritsos@upol.cz, ales.panacek@upol.cz, radek.zboril@upol.cz

D. Panáček, M. Langer, Dr. K. Poláková, Prof. M. Otyepka, Prof. R. Zbořil
Regional Centre of Advanced Technologies and Materials, Czech Advanced Technology and Research Institute, Palacký University Olomouc, Křížkovského 511/8, 779 00 Olomouc, Czech Republic

D. Panáček, L. Hochvaldová, T. Malina, M. Langer, J. Belza, J. Martincová, Dr. A. Panáček
Department of Physical Chemistry, Faculty of Science, Palacký University Olomouc, 17. listopadu 1192/12, 771 46 Olomouc, Czech Republic

Dr. R. Večeřová, Prof. M. Kolář
Department of Microbiology, Faculty of Medicine and Dentistry, Palacký University Olomouc, Hněvotínská 3, 775 15 Olomouc, Czech Republic

Dr. A. Bakandritsos, Prof. R. Zbořil
Nanotechnology Centre, Centre of Energy and Environmental Technologies, VŠB–Technical University of Ostrava, 17. listopadu 2172/15, 708 00 Ostrava-Poruba, Czech Republic

Keywords: antimicrobial, cytocompatibility, silver resistant, graphene

Materials and methods

Chemicals and Reagents. Fluorinated graphite (extent of labelling: >61 wt % F), ammonia (28–30% [w/w], p.a.), sodium borohydride, and sodium citrate dehydrate (p.a.) were obtained from Sigma-Aldrich. Silver nitrate (p.a., Fagron), sodium hydroxide (p.a., Lach-Ner). Sodium thioglycollate (NATG) was purchased from Sigma-Aldrich. Ultrapure water was used (produced

by MERCI AQUAL29) with a conductivity of 0.05 μS cm^{-1}. Graphene oxide (GO) dispersion in water was purchased from Graphenea.

Bacterial Strains. The following standard reference strains (labelling according to the Czech Collection of Microorganism, Masaryk University, Brno, Czech Republic) were used for antimicrobial activity determination: *S. aureus* CCM 4223 (Gram-positive), *E. coli* CCM 3954 (Gram-negative). The following bacterial strains were isolated from human clinical material at the University Hospital, Olomouc, Czech Republic, and used for testing antimicrobial activity: *Staphylococcus epidermidis* (901), methicillin-susceptible *Staphylococcus epidermidis* (Gram-positive), methicillin-resistant *S. aureus* (MRSA), and ESBL-positive *K pneumoniae* (Gram-negative). These strains are part of the collection of microorganisms of the Department of Microbiology (Faculty of Medicine and Dentistry, Palacký University Olomouc) and are stored in cryotubes (ITEST plus, Czech Republic) at –80 °C. *E. coli* and *P. aeruginosa* resistant to silver nanoparticles were provided also by the abovementioned collection of microorganisms of the Department of Microbiology and were prepared as reported previously.[1]

Cell Lines. For cellular toxicity studies, adherent human lung fibroblasts HEL 12469 (ECACC 94101201), human skin fibroblasts BJ (ATCC), and human cervical adenocarcinoma HeLa (ATCC) cell lines were used.

Synthesis of Cyanographene. Cyanographene was prepared according to a previous reported route,[2] but on a larger scale, starting with 4 g of FG. Fluorinated graphite (4 g, *ca*. 129 mmol, based on C-F bonds) was stirred for three days in 240 mL of DMF at room temperature. The resulting dispersion was then divided into six batches of approximately equal volume, in 50 mL

round-bottom glass flasks. These batches were sonicated for 4 h (Bandelin Sonorex, DT 255H type, frequency 35 kHz, power 640 W, effective power 160 W) under a nitrogen atmosphere before being recombined. NaCN (5.1 g, 106 mmol) was then added to the recombined dispersion and the mixture was heated at 130 °C with a condenser under stirring (500 rpm). Both the molar ratio of NaCN to FG and the volume of DMF relative to the mass of FG were lower than in the previous synthesis.[2] After 48 h of stirring, the reaction mixture was separated by centrifugation and purified by successive washing steps using DMF (2×), acetone (3×), ethanol (3×), and water (3×), followed by hot (80 °C) DMF and water. During the centrifugations in DMF and in water, HCl was added (5 wt. %, 0.3 mL) to aid the precipitation of the nitrile-modified graphene flakes. Washings with acidified solvents were also useful for removing Na^+, which was bound to the product as a counter-ion due to the GCN flakes' negative zeta potential. Finally, the product was subjected to dialysis and the final aqueous suspension (pH = ~8, S = ~150 μS cm^{-1}) was stored at room temperature.

Synthesis of GCN/Ag. GCN/Ag was synthesized by chemical reduction of a precursor material, where silver cations were coordinated on the GCN sheet. For this purpose, a 1.5 mL suspension containing 5 mg of GCN was mixed with 10 mL of 2.2×10^{-3} M $AgNO_3$ solution under vigorous stirring for 24 hours at room temperature. Then, the dispersion of the Ag^+-modified GCN was three times purified by washing with distilled water and centrifugation at 15,000 rcf in order to remove silver ions not firmly coordinated on the GCN flakes. After purification, the Ag^+-modified GCN was redispersed in 10 mL of distilled water followed by addition of 10 mL of 3.2×10^{-3} M NH_3 solution and 10 mL of 6.9×10^{-3} M sodium citrate solution. Chemical reduction was initiated by addition of 2 mL of 2.2×10^{-3} M $NaBH_4$ solution and kept in the dark for one

hour. The final silver-nanoparticle-decorated GCN/Ag product was three times washed with distilled water and centrifugated at 10,000 rcf and dried. Dried GCN/Ag was suspended in appropriate volume of water to get a final concentration of 2 g L^{-1} of hybrid for antibacterial and cytotoxicity assays. GCN/Ag contained 13 wt. % of Ag, according to atomic absorption spectroscopy.

Synthesis of Silver Nanoparticles. Colloidal silver 28 nm sized particles were synthesized *via* a modified Tollen's method using maltose as reducing agent, according to Panacek *et al*.[3] Briefly, $[Ag(NH_3)_2]^+$ complex (prepared by mixing $AgNO_3$ with NH_4OH solution) was reacted with maltose at room temperature for 5 min. The initial concentrations of the reaction components were 10^{-3} mol L^{-1} and 0.01 mol L^{-1} for $AgNO_3$ and the reducing agent, respectively. The concentration of ammonia was 0.005 mol L^{-1}.

Silver NPs (size 10 nm) were synthesized using the same method as in the case of 28 nm sized AgNPs but using a stronger reducing agent ($NaBH_4$) instead of maltose and 0.01% gelatin as stabilizer of silver NPs.

Antimicrobial Assays. Antimicrobial activity was evaluated using the standard dilution method, which enables determination of the minimum inhibitory concentration (MIC) of the tested material, for achieving total visible growth inhibition of the tested bacterial strain. The methodology according to the European Committee on Antimicrobial Susceptibility Testing (EUCAST, ref. 40 in the manuscript) was followed. For the purpose of antimicrobial testing, water suspensions of GCN/Ag at the concentration of 2 g L^{-1} were used. Testing was carried out in microtiter plates where the tested samples were diluted by the culture medium (Mueller

Hinton Broth, BD Difco, France) in a geometric progression from 2 to 1024 times. Culture medium was inoculated with the tested bacteria at a concentration of 10^5 to 10^6 CFU mL^{-1}. After 24 h incubation at 37 °C, MIC_{100} values with respect to the concentration of the hybrid, as well as to the concentration of silver only, were determined as the lowest concentrations of the tested substance inhibiting the visible growth of microorganisms (absence of clouding, growth appears as turbidity or as a deposit of cells at the bottom of the well). The obtained Ag-based MIC_{100} values were compared with those of $AgNO_3$ solution and with 28 nm sized AgNPs in water dispersion.

The effect of silver ions on the antibacterial activity of GCN/Ag was evaluated by comparison of the MIC_{100} values of GCN/Ag with and without the addition of a silver ion complexing molecule (thioglycolate, NATG 0.1 w/v). MIC_{100} was tested by the dilution method on the bacterial strain of *E. coli* 5556 in the same way as described previously. After 24 h incubation at 37 °C, MIC_{100} values of GCN/Ag and GCN/Ag^+ NATG were evaluated.

Induction of bacterial resistance towards GCN/Ag nanocomposite. The MIC of the nanocomposite was determined as described above. After the 24-hour cultivation with subinhibitory concentrations of GCN/Ag, 10 µL of Mueller–Hinton broth containing the surviving bacteria were taken from the wells and were sub-cultured on blood agar (TRIOS) at 37 °C for 24 hours. Bacteria grown on blood agar were used for inoculum preparation at a density of 10^6 CFU/mL and for the next culture step. The described procedure by now is considered as one culture step and one bacterial generation. Sixty steps were performed in total in order to monitor any possible increase in the MIC of GCN/Ag, which would indicate the development of bacterial resistance.

Reactive oxygen species (ROS) determination in *E. coli*. Bacteria treated with GCN/Ag for 10 h: Mueller-Hinton (MH) broth containing GCN/Ag at concentration corresponding to MIC_{100} was inoculated in *E. coli* and the bacteria were grown for 8 hours at 37 °C, before the addition of the ROS probe, as described in the following.

Bacteria treated with GCN for 5 h and control samples: *E. coli* was grown to mid-log phase in MH broth at 37 °C and split to three tubes. In the first GCN/Ag was added at concentration corresponding to MIC_{100}. All three samples were incubated for 3 hours at 37 °C, before the addition of the ROS probe, as described in the following.

Detection of ROS: The culture medium from all samples (four in total) was exchanged with PBS and the cultures were transferred to a 96-well microplate. The ROS probe CM-H2DCFDA (Invitrogen, C6827) was added to a final concentration 1 µM. In the case of positive control, hydrogen peroxide was also added to a final concentration 2 mM. The samples were incubated at 37 °C for another 2 hours in the dark. Finally, the fluorescence was recorded using fluorescence microplate reader Infinite 200 Pro (Tecan) at ex./em. wavelengths of 492 nm/527 nm. The procedure was performed 5 times.

Cell Cultures and Cytotoxicity/Cell Viability Assay. For cellular toxicity, human lung fibroblasts HEL 12469 and human skin fibroblasts BJ (ATCC) cells were cultivated at 37 °C under a 5% CO_2 atmosphere in EMEM—Eagle's Minimum Essential Medium (Sigma-Aldrich), enriched by L-Glutamine, non-essential amino acids (NEAA), fetal bovine serum (FBS), PenStrep (5000 U penicillin, 5 mg streptomycin mL^{-1}), and sodium bicarbonate (7.5%). The HeLa cells were cultivated at 37 °C under a 5% CO_2 atmosphere in DMEM – Dulbecco's modified Eagle's medium (Invitrogen, USA) supplemented with L-Glutamine, 10% FBS, and 1% PenStrep (10000 U penicillin, 10 mg streptomycin mL^{-1}).

Cell viability was evaluated using a BD FACSVerse flow cytometer (BD Biosciences, USA). Ten thousand cells were seeded per well in a 96-well plate. Cells were incubated with GCN/Ag at various concentrations (based on the total mass) from 8 to 120 mg L^{-1} for 24 h. After 24 h, the supernatant was collected, and cells were gently washed with PBS solution (0.1 M, 7.4 pH). Then, cells were detached with trypsin (0.25% in EDTA, Sigma-Aldrich), resuspended in 100 µL of culture media and added to supernatant. Viability of cells was determined by propidium iodide (PI) and calcein-AM fluorescent probes. Cells were incubated with 1 µL of PI (1 µg mL^{-1}) and 2 µL of calcein-AM, diluted in DMSO (50 µM), for 20 minutes and the fluorescent signal was measured by flow cytometer using red channel (exc. 488/em. 586) for PI and green channel (exc. 488/em. 527) for calcein. Red signal of PI revealed dead cells, which lost their membrane integrity, while green signal was represented by cells with active intracellular esterases that catalyzed the non-fluorescent calcein-AM to highly fluorescent green calcein. The viability was established and normalized to control cells with 100% viability.

Instrumentation. The materials were characterized by transmission electron microscopy (TEM) using a JEM 2010 TEM instrument (Jeol, Japan). UV-vis spectra of GCN/Ag dispersions were recorded on a Specord S 600 (Analytic Jena, Germany) spectrophotometer. Zeta potential of the GCN/Ag colloid was obtained by electrophoretic mobility measurements using Zetasizer NanoZS (Malvern, UK). The amount of silver immobilized on GCN, as well as the amount of silver released from GCN/Ag during leaching tests was measured using atomic absorption spectroscopy (AAS), on a ContrAA 600 with graphite furnace (Analytik Jena AG, Germany) equipped with a high-resolution Echelle double monochromator (spectral band width, 2 pm at 200 nm) and a xenon lamp as a continuum radiation source. For AAS measurements, GCN/Ag

was added (at concentration equal to 0.01g L^{-1}) into a solution of nitric acid (2 % w/w) and sonicated for 10 minutes in order to quantitatively dissolve silver. In order to determine leaching of AgNPs from GCN/Ag, 5.8 mg of GCN/Ag was dispersed in 4 mL of distilled water and 4 mL of culture medium followed by shaking for 24 and 72 hours. Then it was filtered using syringe filter (0.2 μm pore size) to obtain the possibly released AgNPs and/or leached silver as Ag cations. After filtration, in order to dissolve the possibly present Ag NPs, 2 mL of filtrate were put to 50 mL of 2% nitric acid for 30 min. The quantity of released silver in the filtrate was determined with AAS.

FTIR spectra were recorded on an iS5 FTIR spectrometer (Thermo Nicolet) using the Smart Orbit ZnSe ATR accessory. Briefly, a droplet of an ethanol dispersion of the relevant material was placed on the ZnSe crystal and dried. The spectra were then acquired by summing 52 scans while using a nitrogen gas flow through the ATR accessory. ATR and baseline correction were applied to the collected spectra.

Raman spectra were recorded on a DXR Raman microscope using the 633 nm excitation line of a diode laser. For measuring 1.5 mg of GCN/Ag was diluted in 4 mL of distilled water.

High-resolution X-ray photoelectron spectroscopy (HR-XPS) was carried out with a PHI VersaProbe II (Physical Electronics) spectrometer using an Al K_α source (15 kV, 50 W). The obtained data were evaluated and deconvoluted with the MultiPak (Ulvac - PHI, Inc.) software package. The spectral analysis process involved Shirley background subtraction and peak deconvolution using mixed Gaussian–Lorentzian functions.

HR-TEM images were obtained using a HR-TEM TITAN 60-300 microscope with an X-FEG type emission gun, operating at 300 kV. Scanning transmission electron microscopy high-angle annular dark-field imaging (STEM-HAADF) analysis for EDS (energy-dispersive X-ray

spectroscopy) elemental mapping on the products was performed with a FEI Titan HR-TEM microscope operating at 80 kV. For this analysis, a droplet of an aqueous dispersion of the material under study with a concentration of ~0.1 mg mL^{-1} was deposited on a carbon-coated copper grid and dried at room temperature for 24 hours.

SEM Observation of Bacteria. Bacteria were incubated in normal bacteria cultivation medium or in presence of GCN/Ag. Bacteria were incubated for 24 h in 96-well microtiter plates in the same way as described above (standard dilution method). Sub-inhibitory concentration of the GCN/Ag was used in order to observe bacteria before their death, since at higher concentration, no bacteria could be observed, apart from bacterial debris. In the following, bacteria were washed from broth residues by centrifugation, redispersion in PBS, repeated centrifugation and finally were redispersed in water. Then bacteria were fixed on gold-coated microscope glass slides by flame, in order to be characterized by scanning electron microscopy. All the samples prepared by this approach were observed by scanning electron microscope (Hitachi SU6600) with acceleration voltage 1.5 kV.

Computations. All DFT calculations were performed using projector-augmented plane method, implemented in Vienna Ab initio Simulation Package (VASP).[4,5] GCN (cyanographene) was modelled as a 3×3 and 4×4 supercell of graphene; its surface was covered by 10% of CN groups. In both, at least 15 Å of vacuum was set along the z-axis to avoid the spurious interaction due to periodic boundary conditions. The energy cutoff was set to 600 eV and the Brillouin zone was sampled by 5×5×1 *k*-points. The geometric optimization of the model and adsorption energies were calculated using the optimized van der Waals functional optB86b-vdW, which properly

includes weak van der Waals bonding and provides reliable adsorption energies.[6] The ultra-small silver NPs were represented as a) the single silver atom or b) the icosahedral nanocluster comprising of 13 Ag atoms. The binding in GCN/Ag was assessed by calculating the adsorption energies of AgNP models to the GCN support using Equation (1):

$$E_{ads} = E_{\mathrm{GCN+AgNP}} - E_{\mathrm{GCN}} - E_{\mathrm{AgNP}} \quad (1)$$

in which $E_{\mathrm{GCN+AgNP}}$ denotes the total energy of GCN/Ag, *i.e.*, silver NP adsorbed represented by the silver atoms or icosahedral nanocluster attached to the CN group of cyanographene. E_{GCN} is the total energy of cyanographene and E_{AgNP} is the total energy of the isolated silver atom or icosahedral nanocluster.

The Ag-N bond was characterized by calculating the Mulliken and Hirshfeld charges and electron localization function (ELF) for the GCN/Ag. The Mulliken and Hirshfeld charges were calculated in the FHI-aims code[7] using the cluster model of GCN/Ag. The ELF was calculated in VASP and the figure of the ELF density was plotted using the VESTA package.[8]

The Raman shift of the Ag-N stretching vibration was obtained by calculating the vibrational spectrum of GCN/Ag and consequent analysis of the eigenvectors of the force-constant matrix. The elements of the force-constant matrix were determined using density-functional perturbation theory in VASP.

Classical all-atom molecular dynamics (MD) simulations were performed using the Gromacs 5.0 software, AMBER Lipid14 and AMBER ff99 force field.[9–11] Lennard-Jones (LJ) parameters for the cyanographene carbon atoms were taken from the literature.[12] The LJ parameters developed for simulations of silver nanoclusters in water were adopted, and the LJ parameters for interaction of silver and carbons atoms were taken from the literature, too.[13,14] The silver atoms and hence the entire nanoclusters were modelled as uncharged. Partial atomic charges of

cyano-group and the C–H edges of cyanographene and graphene were derived using RESP method, the aromatic carbon atoms were uncharged.[15] The model of face centered cubic interaction (fcc) silver nanoclusters were built using NanoCrystal,[16] where two common crystal planes (111) and (100) were chosen to form the final structure having 55 atoms (diameter ~5.8 Å). The model of GCN/Ag was built by grafting two silver nanoclusters on cyanographene flakes (*i.e.*, graphene flake having ~13.5% degree of functionalization by cyano groups of size ~42.2 × 43.5 Å) each via one Ag–N bond. The bonded parameters were set based on our ab-initio calculations.

Real cell walls of Gram-negative and Gram-positive bacteria have very complex composition.[17] and their modeling still represents a challenge for computational chemistry. The bacterial cell wall of Gram-positive bacteria involves thick layer of peptidoglycan network, which models are still under intensive development.[18,19] The bacterial cell wall of Gram-negative bacteria contains an outer membrane with lipopolysaccharides and a thin peptidoglycan layer. Both bacteria have inner plasma membrane containing 60/13 % of phosphatidylethanolamine (PE) and 13/77 % of phosphatidylglycerol (PG) (Gram-negative/Gram-positive).[20,21] The results of MD simulations should be interpreted with care taking into account that homogeneous membrane models cannot cover all the complexity and diversity of the bacterial membranes.[22] We used a simplified model based on 1-palmitoyl-2-oleoyl-sn-glycero-3-phosphoglycerol (POPG) as a dominant lipid in bacterial membrane of Gram-positive bacteria. POPG membrane was built as a bilayer of 128 POPG molecules by CHARMM-GUI,[23] and pre-equilibrated for 85 ns. After a minimization, GCN/Ag was placed together with the POPG bilayer in a cubic box (~66 × 66 × 110 Å). The entire simulation box was solvated with TIP3P water model containing 0.154 M NaCl using Amber-adapted Åqvist parameters for Na^+ and Smith & Dang parameters for Cl^-.[24–26] Periodic

boundary conditions were applied in all three dimensions, bonds involving hydrogens were constrained using the LINCS algorithm,[27] allowing a 2 fs time step in all simulations. The cutoff radius for van der Waals interactions and the real part of electrostatics was set to 10 Å. Long-range electrostatic interactions were treated using the particle-mesh Ewald (PME) method. A long-range dispersion correction was applied to the energy and pressure. The productive runs were carried out in an NpT ensemble for 1.0 µs. The temperature was held at 310 K using Nose-Hoover thermostat with a 0.5 ps coupling constant and coupled with the semi-isotropic barostat with a 1.0 coupling constant and a reference pressure of 1.0 bar.[28–30] Figures were rendered using PyMOL software.[31] Comparative simulations with the same methodological workflow were carried out for mixed POPE (1-palmitoyl-2-oleoyl-sn-glycero-3-phosphoethanolamine):POPG (3:1) membrane with GCN/Ag hybrid, two silver nanoclusters, cyanographene and graphene nanoflakes.

Statistical analysis

For human cell studies as well as for bacterial ROS determination, we performed three and five independent experiments respectively, and the mean ± standard deviation (SD) was calculated. Student t-test was performed for each concentration and compared with negative control using the statistical software TIBCO, 2018. Any difference was considered significant and very significant at $p \leq 0.05$ and $p \leq 0.01$, respectively.[32]

Supplementary Tables and Figures.

Table S1. MIC values (for 100% bacterial inhibition growth) and cytocompatibility concentrations of the GCN/Ag hybrid compared with representative graphene/Ag-based antibacterial agents reported in the literature.

Ref.	MIC[a] [mg L^{-1}] [a]		Incubation time [h]	Cytocompatibility [mg L^{-1}] [a]	Cell line (strain)	Size [nm]
	Normal strains	Drug-res. bacteria				
**GCN/Ag**	**1.8 (0.2)**	**3.7 (0.5)**	**24**	**60 (7.5)**	**human lung fibroblasts (HEL 12469)**	**4–8**
[33]	2 (0.6)	5 (1.4)	18	10 (2.8) 85.5%	cancer cell (HeLa)	5–10
[34]	0.7 (0.35)	X	Overnight	3 (1.5) 80%	mouse macrophage (RAW 264.7)	10
[35]	1.6	X	24	1.2	human embryonic kidney (HEK 293)	22
[36]	10 (5) 94%	X	Overnight	10 (5) 85%	cancer cell (HeLa)	30–50
[37]	2 (1)	2 (1) 80%	15	4(2)	cancer cells (HeLa, MCF-7)	2–10
[38]	50 (1.7)	X	18	X		30–50
[39]	3.2 (1.6)	X	24	X		14
[40]	2	X	8	X		5–15
[41]	X	2.4 (1.1)	24	X		10–30
[42]	60	X	24	X		8
[43]	62.5 (43.7)	X	24	X		1–15

[a] Numbers refer to values with respect to the mass of the hybrids and the numbers in parentheses refer to the values with respect to the mass of the Ag only, according to its content in the hybrid. The percentiles appearing bellow refer to cases where the bacterial growth inhibition was not 100%, or where cytocompatibility did not refer to 100% viability of the cells. “X” stands for unavailability of data.

Table S2. MIC values (for 100% bacterial inhibition growth) of the starting GCN, GCN/Ag hybrid, ionic silver ($AgNO_3$), GO, and colloidal AgNPs against drug-sensitive, multidrug-resistant, and AgNP-resistant bacterial strains.

Material / Bacterial strain	GCN/Ag[a]	GCN	GO	$AgNO_3$	AgNPs (28 nm)	AgNPs (10 nm)
Escherichia coli CCM 3954 (Gram-negative)	1.8 (0.2)	>1880	>1500	0.8	3.4	1.7
Staphylococcus aureus CCM3953 (Gram-positive)	14.7 (1.8)	>1880	>1500	1.7	3.4	1.7
Staphylococcus epidermidis (901) (Gram-positive)	59.7 (7.2)	>1880	>1500	0.8	1.7	0.8
Staphylococcus aureus (MRSA 4591) (Gram-positive)	14.7 (1.8)	>1880	>1500	0.8	6.8	1.7
Klebsiella pneumoniae (ESBL 2486) (Gram-negative)	3.7 (0.4)	>1880	>1500	1.7	6.8	3.4
Escherichia coli CCM (AgNP-resistant 3954) (Gram-negative)	27.6 (3.6)	>1880	>1500	1.7	108	108
Pseudomonas aeruginosa (AgNP-resistant 008) (Gram-negative)	14.7 (1.8)	>1880	>1500	1.7	54	54

[a] Values refer to the mass of GCN/Ag and values in parentheses refer to the MIC values with respect to the mass of the Ag only, according to its content in the hybrid. The Ag content in GCN/Ag hybrid was 13 wt. %.

Table S3. MIC values (for 100% bacterial inhibition growth) of the GCN/Ag hybrid compared with representative antibacterial agents reported in the literature.

Material	Incubation time [h]	MIC[a] [mg L^{-1}]		Ref.
		E. coli	MR *S. aureus*	
**GCN/Ag**	**24**	**1.8 (0.2)[a]**	**14.7 (1.9)[a]**	**This work**
**Ho-GO-Au**	Overnight	X	9.8<br>50%	[44]
**AgMOF-N1**	24	4	X	[45]
**GMO-LL-37**	X	40	inactive	[46]
**SCG**	11	X	120<br>90%	[47]
**CS - DA/PMB**	12	2		[48]
**Si sheets**	X	200	200	[49]
**OSiNDs - Van**	48	X	5<br>100%	[50]
**CaO_2**	24	100	X	[51]
**Ag@CD-MOF**	10	32	512	[52]
**Cu_2WS_4**	4	1.7	0.7	[53]
**Pd@Ir**	12	25<br>~60%	X	[54]

[a] Numbers refer to values with respect to the mass of the GCN/Ag hybrid and the numbers in parentheses refer to the values with respect to the mass of the Ag only, according to its content in the hybrid. The percentiles appearing bellow refer to cases where the bacterial growth inhibition was not 100%, or where cytocompatibility did not refer to 100% viability of the cells. "X" stands for unavailability of data.

Table S4. MIC_{100} values for the hybrid (GCN/Ag), silver nanoparticles (28 nm diameter, AgNPs), and free silver ions ($AgNO_3$ solution) in presence of the Ag^+-complexing agent of sodium thioglycollate (NATG).

Antibacterial agent	Minimum inhibitory concentration (mg L^{-1})[a]		
	**CONTROL**	**+NATG**	**MIC_{100} increase**
GCN/Ag	0.8	3.4	4×
AgNPs	3.4	27	8×
$AgNO_3$	0.8	13.5	16×

[a] Experiments performed in triplicates.

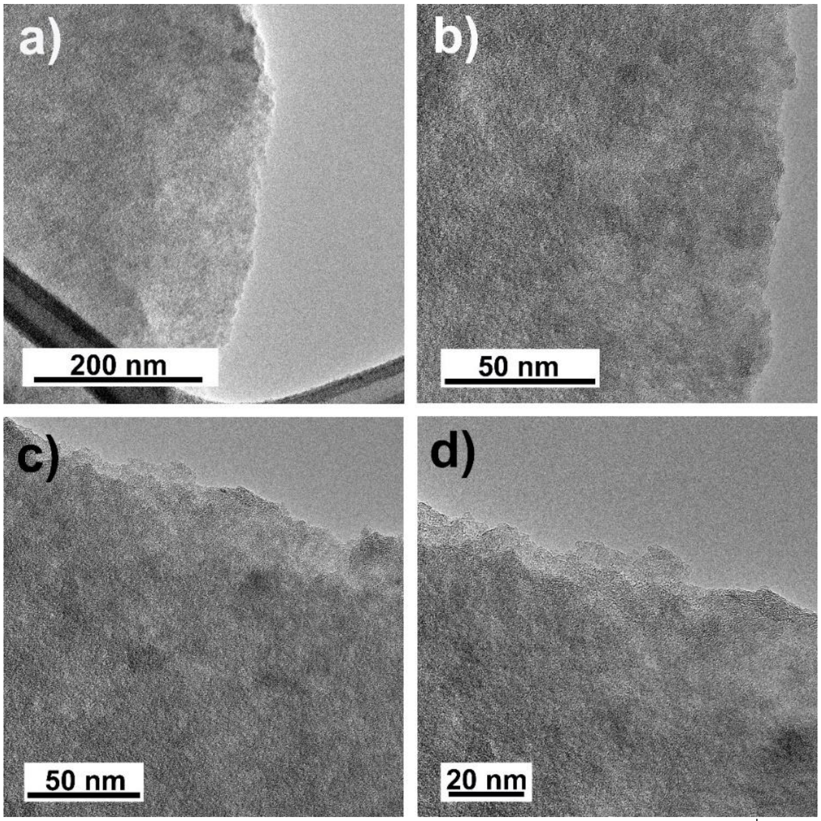

Figure S1. a–d) HRTEM images of the ionic GCN/Ag^+.

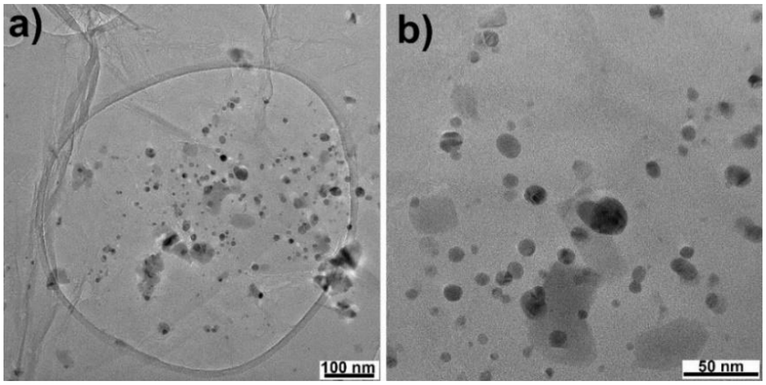

Figure S2. a,b) TEM images of AgNP-decorated graphene oxide prepared under identical conditions as the GCN/Ag material.

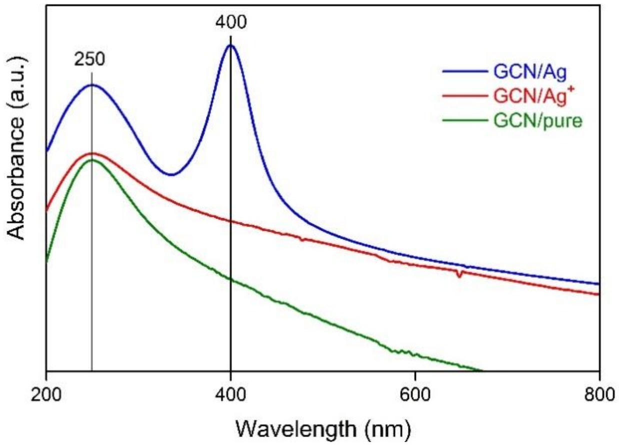

Figure S3. UV-Vis absorption spectra of pure GCN, GCN with immobilized silver in ionic form (GCN/Ag^+) and of GCN with immobilized AgNPs.

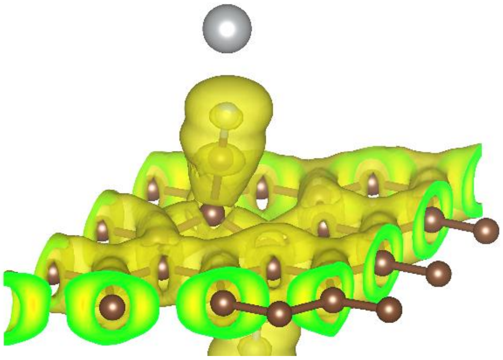

Figure S4. Electron localization function plot of the GCN/Ag^+ precursor for the evaluation of the Ag-N bond character.

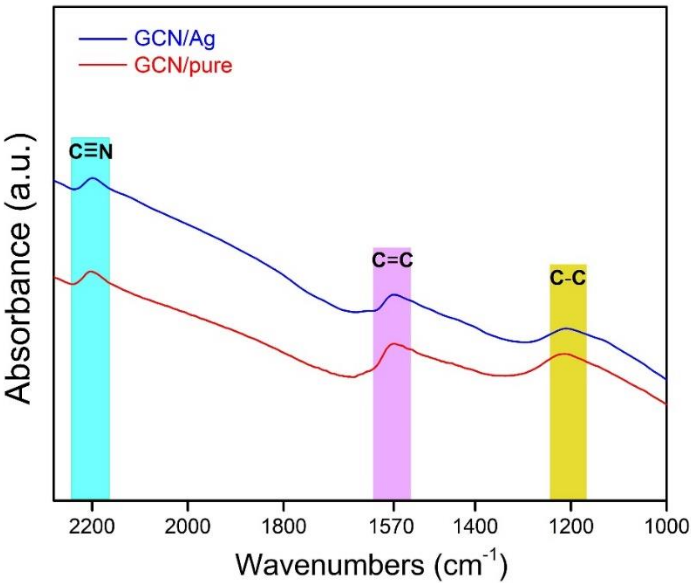

Figure S5. FT-IR spectra of pure GCN and GCN/Ag nanohybrid. During the Ag ions reduction reaction, no changes on the nitrile groups took place, as evidenced by the persistent band at 2200 cm^{-1}.

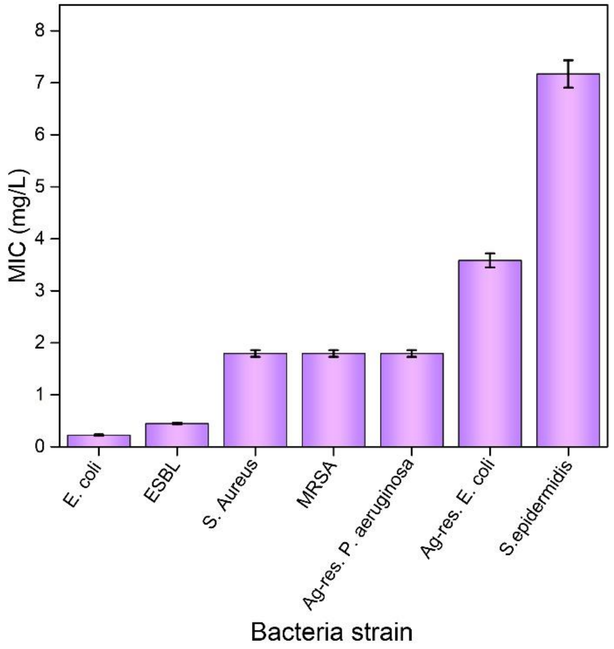

Figure S6. MIC_{100} values with error bars of GCN/Ag hybrid against drug-sensitive, multidrug-resistant, and AgNP-resistant bacterial strains. The error bars originate from the respective error in the Ag content in the different GCN/Ag batches and from the measurement method. (n = 3)

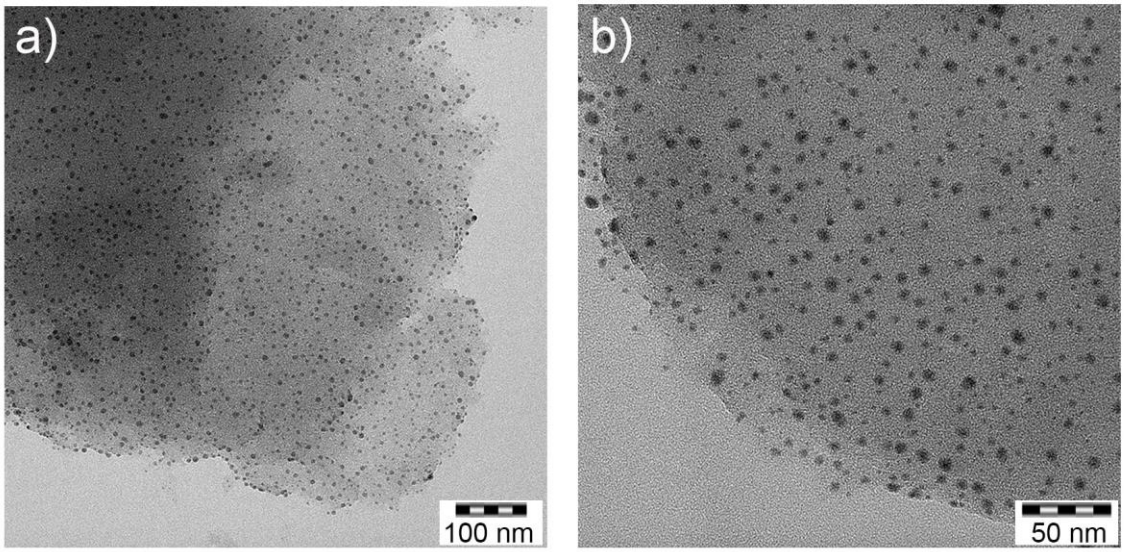

FigureS7. a,b) TEM images of GCN/Ag after 6 months of storage in water.

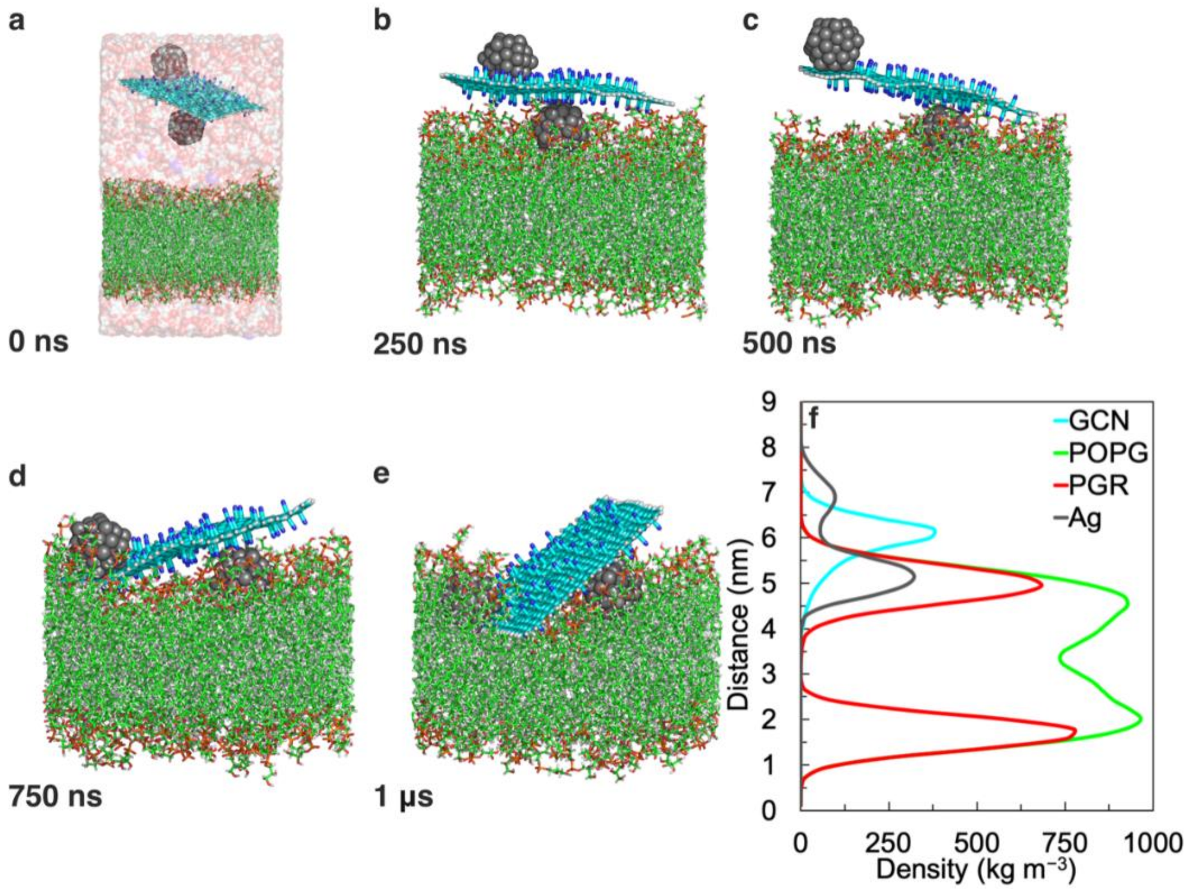

Figure S8. a-e) Time evolution of GCN/Ag during its interaction with phospholipid POPG (1-palmitoyl-2-oleoyl-sn-glycero-3-phosphoglycerol) membrane studied by MD simulation (color coding: grey – silver; cyan and green – carbon; red – oxygen; blue – nitrogen; orange – phosphorus; water molecules and ions are omitted for clarity, except for the starting structure, 0 ns); and f) the corresponding density profile (averaged over last 800 ns of the MD simulation) along the normal to the lipid bilayer plane displaying densities of the entire POPG membrane (green), polar lipid headgroups of phosphatidylglycerols (red, PGR), silver nanoclusters (grey), cyanographene GCN (cyan).

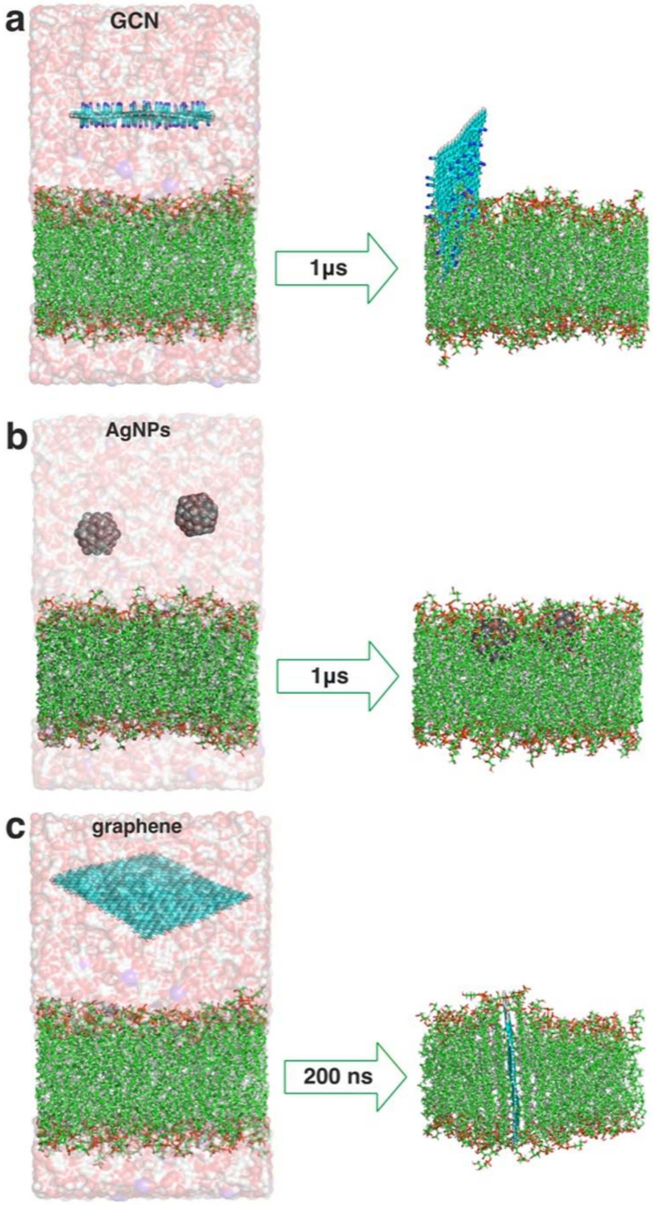

Figure S9. Snapshots form MD simulations of a) cyanographene, b) two silver nanoclusters, and c) graphene on POPG membrane (color coding: grey – silver; cyan and green – carbon; red – oxygen; blue – nitrogen; orange – phosphorus. For the final structures, water and ions are omitted for clarity).

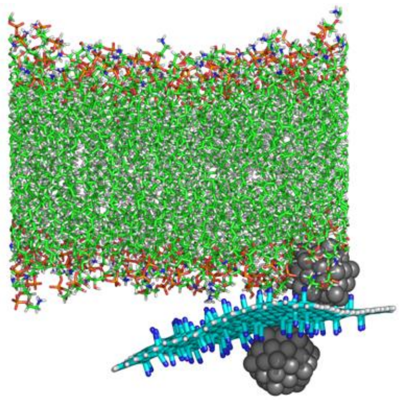

Figure S10. The interaction of GCN/Ag with POPE:POPG (3:1) membrane studied by MD simulations (color coding: grey – silver; cyan and green – carbon; red – oxygen; blue – nitrogen; orange – phosphorus; water molecules and ions are omitted for clarity).

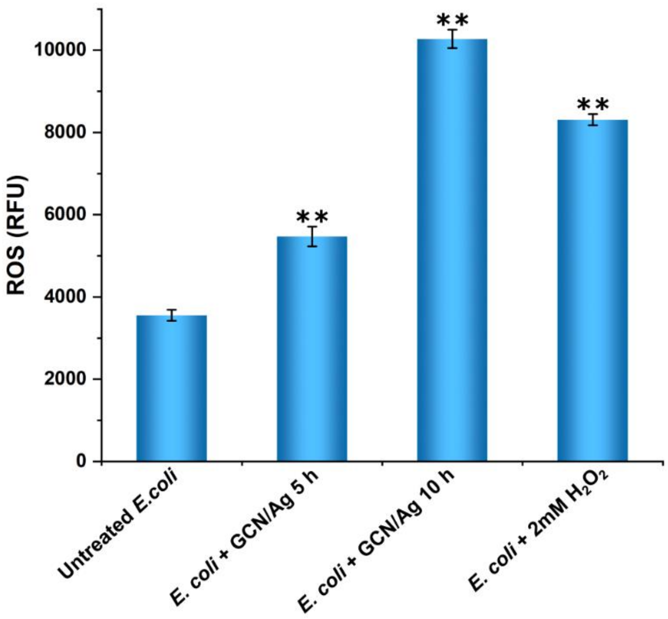

Figure S11. Generation of reactive oxygen species (ROS) in *E. coli* without any treatment or treated with GCN/Ag for 5 and 10 hours at a concentration corresponding to MIC_{100}. Hydrogen peroxide (2mM, 2h of incubation) was used as positive control. Error bars represent ±SD, n = 5. ** indicates p-value < 0.01.

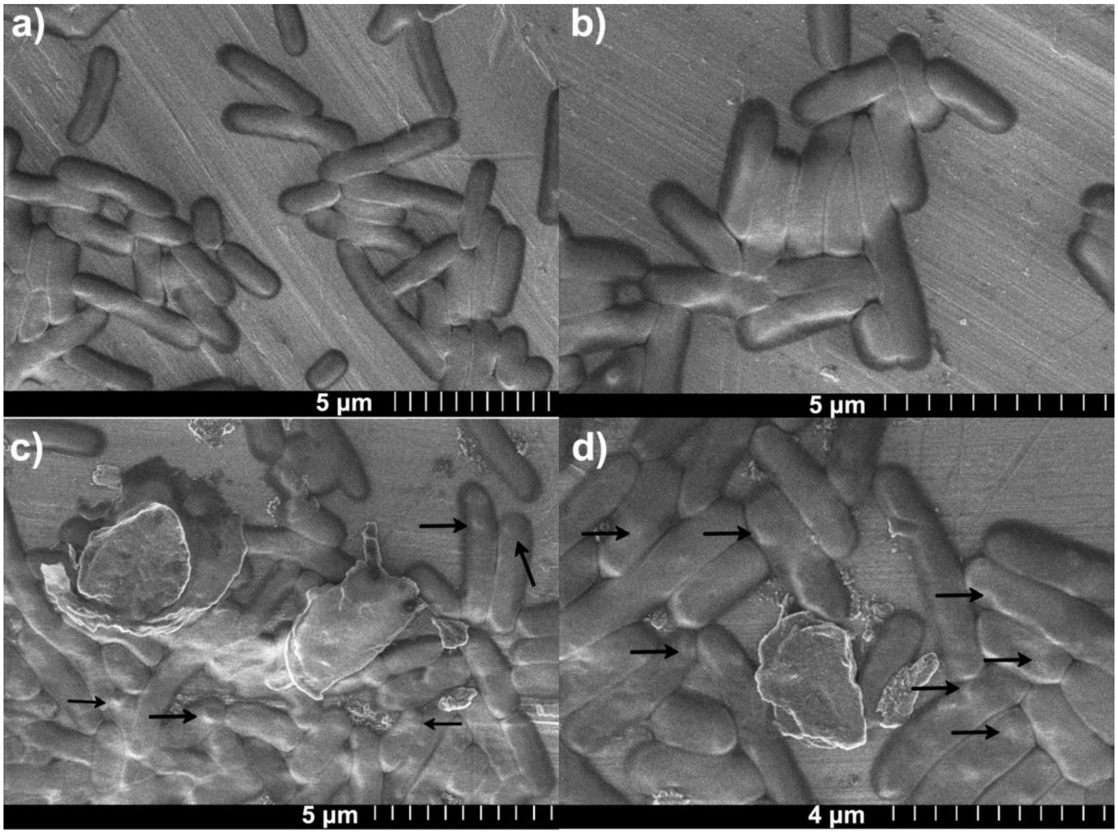

Figure S12. a,b) Native Ag-resistant *E. coli* and c,d) treated with GCN/Ag at sub-inhibitory concentration. The native *E. coli* show smooth cell membrane surface, while those treated with GCN/Ag show quite increased roughness or bumps, indicated in some cases with the arrows. Bacteria were treated with 1.7 μg_{Ag} mL^{-1}.

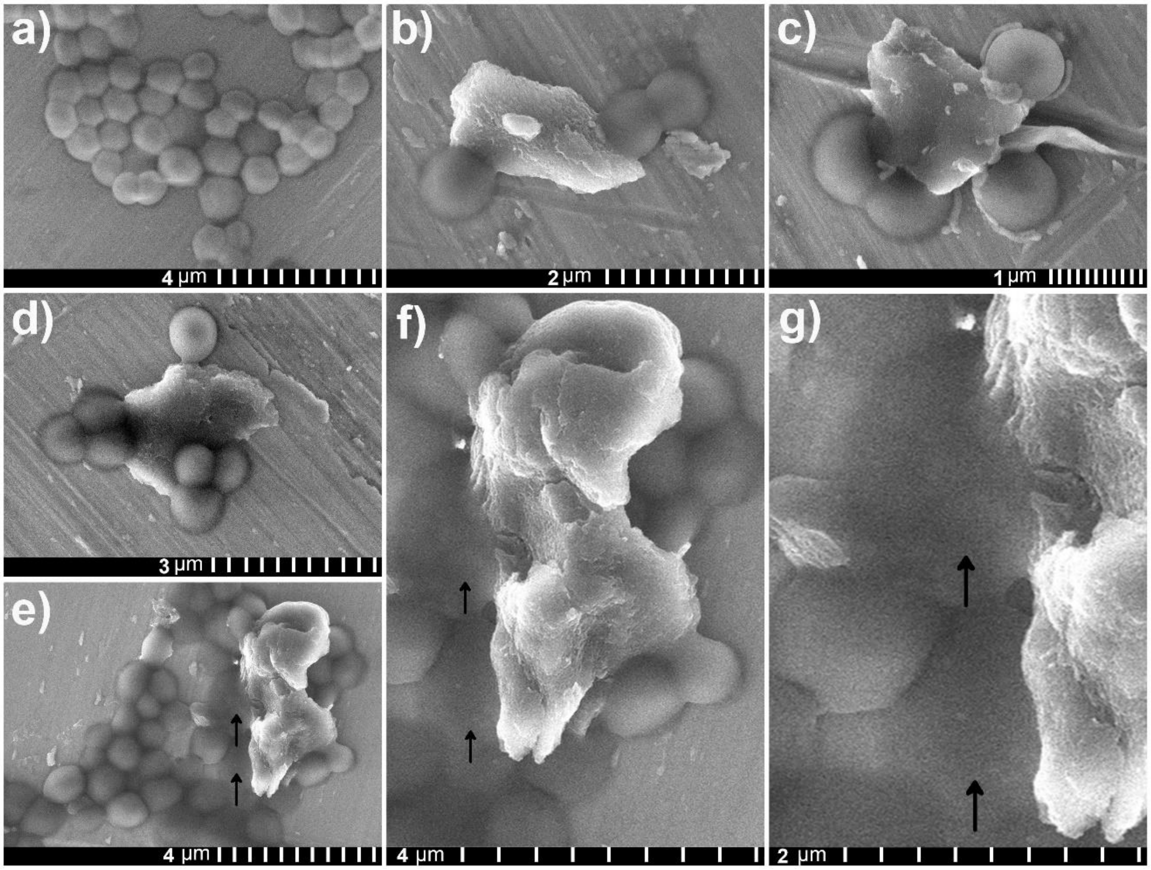

Figure S13. a) Native MRSA and b–e) treated with GCN/Ag at sub-inhibitory concentration. f,g) Details of MRSA treated with GCN/Ag at sub-inhibitory concentration. The arrows show biological material corresponding to dead MRSA bacteria cells. MRSA were treated with 13.5 μg_{Ag} mL^{-1}; in general there are no evident membrane changes in this case of the Gram-positive bacteria. Nevertheless, GCN/Ag remained very effective in this case as well.

REFERENCES

[1] A. Panáček, L. Kvítek, M. Smékalová, R. Večeřová, M. Kolář, M. Röderová, F. Dyčka, M. Šebela, R. Prucek, O. Tomanec, R. Zbořil, *Nat. Nanotechnol.* **2018**, *13*, 65.

[2] A. Bakandritsos, M. Pykal, P. Boński, P. Jakubec, D. D. Chronopoulos, K. Poláková, V. Georgakilas, K. Čépe, O. Tomanec, V. Ranc, A. B. Bourlinos, R. Zbořil, M. Otyepka, *ACS Nano* **2017**, *11*, 2982.

[3] Aleš Panáček, Libor Kvítek, Robert Prucek,Milan Kolář, Renata Večeřová, Naděžda Pizúrová, Virender K. Sharma, Tat'jana Nevěčná, Radek Zbořil, *J. Phys. Chem. B* **2006**, *110*, 16248.

[4] P. E. Blöchl, *Phys. Rev. B* **1994**, *50*, 17953.
[5] G. Kresse, *Phys. Rev. B* **1999**, *59*, 1758.
[6] P. Lazar, F. Karlický, P. Jurečka, M. Kocman, E. Otyepková, K. Šafářová, M. Otyepka, *J. Am. Chem. Soc.* **2013**, *135*, 6372.
[7] V. Blum, R. Gehrke, F. Hanke, P. Havu, V. Havu, X. Ren, K. Reuter, M. Scheffler, *Comput. Phys. Commun.* **2009**, *180*, 2175.
[8] K. Momma, F. Izumi, *J. Appl. Crystallogr.* **2011**, *44*, 1272.
[9] D. V. D. Spoel, E. Lindahl, B. Hess, G. Groenhof, A. E. Mark, H. J. C. Berendsen, *J. Comput. Chem.* **2005**, *26*, 1701.
[10] Å. A. Skjevik, B. D. Madej, C. J. Dickson, C. Lin, K. Teigen, R. C. Walker, I. R. Gould, *Phys. Chem. PCCP* **2016**, *18*, 10573.
[11] J. Wang, P. Cieplak, P. A. Kollman, *J. Comput. Chem.* **2000**, *21*, 1049.
[12] A. Cheng, W. A. Steele, *J. Chem. Phys.* **1990**, *92*, 3858.
[13] A. Manoranjan, S. Jha, *BioEn*, **2018**, 5, 169.
[14] M. Neek-Amal, R. Asgari, M. R. Rahimi Tabar, *Nanotechnology* **2009**, *20*, 135602.
[15] C. I. Bayly, P. Cieplak, W. Cornell, P. A. Kollman, *J. Phys. Chem.* **1993**, *97*, 10269.
[16] A. Chatzigoulas, K. Karathanou, D. Dellis, Z. Cournia, *J. Chem. Inf. Model.* **2018**, 58, 12, 2380–2386.
[17] T. J. Silhavy, D. Kahne, S. Walker, *Cold Spring Harb. Perspect. Biol.* **2010**, *2*, a000414.
[18] S. Khalid, T. J. Piggot, F. Samsudin, *Acc. Chem. Res.* **2019**, *52*, 180.
[19] R. Vaiwala, P. Sharma, M. Puranik, K. G. Ayappa, *J. Chem. Theory Comput. 2020, 16, 8, 5369–5384.*
[20] D. E. Warschawski, A. A. Arnold, M. Beaugrand, A. Gravel, É. Chartrand, I. Marcotte, *Biochim. Biophys. Acta BBA - Biomembr.* **2011**, *1808*, 1957.
[21] N. Malanovic, K. Lohner, *Biochim. Biophys. Acta BBA - Biomembr.* **2016**, *1858*, 936.
[22] E. R. Rojas, G. Billings, P. D. Odermatt, G. K. Auer, L. Zhu, A. Miguel, F. Chang, D. B. Weibel, J. A. Theriot, K. C. Huang, *Nature* **2018**, *559*, 617.
[23] S. Jo, T. Kim, V. G. Iyer, W. Im, *J. Comput. Chem.* **2008**, *29*, 1859.
[24] W. L. Jorgensen, J. Chandrasekhar, J. D. Madura, R. W. Impey, M. L. Klein, *J. Chem. Phys.* **1983**, *79*, 926.
[25] J. Åqvist, *J. Phys. Chem.* **1990**, *94*, 8021.
[26] D. E. Smith, L. X. Dang, *J. Chem. Phys.* **1994**, *100*, 3757.
[27] B. Hess, H. Bekker, H. J. C. Berendsen, J. G. E. M. Fraaije, *J. Comput. Chem.* **1997**, *18*, 1463.
[28] S. Nosé, *Mol. Phys.* **1984**, *52*, 255.
[29] W. G. Hoover, *Phys. Rev. A* **1985**, *31*, 1695.
[30] H. J. C. Berendsen, J. P. M. Postma, W. F. van Gunsteren, A. DiNola, J. R. Haak, *J. Chem. Phys.* **1984**, *81*, 3684.
[31] Schrodinger LLC. The PyMOL Molecular Graphics System, Version 1.8., **2015**.
[32] *TIBCO Software Inc. Statistica (Data Analysis Software System), Version 13. Http://Tibco.Com.*, **2018**.
[33] R. Zhao, M. Lv, Y. Li, M. Sun, W. Kong, L. Wang, S. Song, C. Fan, L. Jia, S. Qiu, Y. Sun, H. Song, R. Hao, *ACS Appl. Mater. Interfaces* **2017**, *9*, 15328.
[34] S. Kellici, J. Acord, A. Vaughn, N. P. Power, D. J. Morgan, T. Heil, S. P. Facq, G. I. Lampronti, *ACS Appl. Mater. Interfaces* **2016**, *8*, 19038.

[35] W. Shao, X. Liu, H. Min, G. Dong, Q. Feng, S. Zuo, *ACS Appl. Mater. Interfaces* **2015**, *7*, 6966.
[36] J. Tang, Q. Chen, L. Xu, S. Zhang, L. Feng, L. Cheng, H. Xu, Z. Liu, R. Peng, *ACS Appl. Mater. Interfaces* **2013**, *5*, 3867.
[37] S. Chen, Y. Quan, Y.-L. Yu, J.-H. Wang, *ACS Biomater. Sci. Eng.* **2017**, *3*, 313.
[38] H.Z. Zhang , C. Zhang , G.M. Zeng , J.L Gong , X.M. Ou , S.Y. Huan, *J. Colloid Interface Sci.* **2016**, *9*, 40.
[39] Z. Zhu, M. Su, L. Ma, L. Ma, D. Liu, Z. Wang, *Talanta* **2013**, *117*, 449.
[40] L. Yu, Y. Zhang, B. Zhang, J. Liu, *Sci. Rep.* **2014**, *4*, 4551.
[41] B. Marta, M. Potara, M. Iliut, E. Jakab, T. Radu, F. Imre Lucaci, K. Gabriel, O. Popescu, S. Astilean, *Colloids Surf. Physicochem. Eng. Asp.* **2015**, *487*, 113.
[42] S. Fathalipour, M. Mardi, *Mater. Sci. Eng. C* **2017**, *79*.
[43] R. G. Bai, K. Muthoosamy, F. N. Shipton, A. Pandikumar, P. Rameshkumar, N. M. Huang, S. Manickam, *RSC Adv.* **2016**, *6*, 36576.
[44] K. Zheng, K. Li, T.-H. Chang, J. Xie, P.-Y. Chen, *Adv. Funct. Mater.* **2019**, *29*, 1904603.
[45] N. A. Travlou, M. Algarra, C. Alcoholado, M. Cifuentes-Rueda, A. M. Labella, J. M. Lázaro-Martínez, E. Rodríguez-Castellón, T. J. Bandosz, *ACS Appl. Bio Mater.* **2018**, *1*, 693.
[46] M. Zabara, B. Senturk, M. Gontsarik, Q. Ren, M. Rottmar, K. Maniura-Weber, R. Mezzenga, S. Bolisetty, S. Salentinig, *Adv. Funct. Mater.* **2019**, *29*, 1904007.
[47] H. Han, J. Zhu, D.-Q. Wu, F.-X. Li, X.-L. Wang, J.-Y. Yu, X.-H. Qin, *Adv. Funct. Mater.* **2019**, *29*, 1806594.
[48] M. Chai, Y. Gao, J. Liu, Y. Deng, D. Hu, Q. Jin, J. Ji, *Adv. Healthc. Mater.* **2020**, *9*, 1901542.
[49] Y. Luo, M. Ge, H. Lin, R. He, X. Yuan, C. Yang, W. Wang, X. Zhang, *Adv. Healthc. Mater.* **2020**, *9*, 1901375.
[50] X. Chen, X. Zhang, F. Lin, Y. Guo, F.-G. Wu, *Small* **2019**, *15*, 1901647.
[51] S. Shen, M. Mamat, S. Zhang, J. Cao, Z. D. Hood, L. Figueroa-Cosme, Y. Xia, *Small* **2019**, *15*, 1902118.
[52] S. Shakya, Y. He, X. Ren, T. Guo, A. Maharjan, T. Luo, T. Wang, R. Dhakhwa, B. Regmi, H. Li, R. Gref, J. Zhang, *Small* **2019**, *15*, 1901065.
[53] J. Shan, X. Li, K. Yang, W. Xiu, Q. Wen, Y. Zhang, L. Yuwen, L. Weng, Z. Teng, L. Wang, *ACS Nano* **2019**, *13*, 13797.
[54] T. Cai, G. Fang, X. Tian, J.-J. Yin, C. Chen, C. Ge, *ACS Nano* **2019**, *13*, 12694.
